# Peroxisome Proliferator-Activated Receptor β/δ: A Link Between Metabolism, Inflammation, and Fibrosis in Metabolic Dysfunction-Associated Steatotic Liver Disease

**DOI:** 10.3390/cells15050464

**Published:** 2026-03-05

**Authors:** Xavier Palomer, Jue-Rui Wang, Xiaoman Tang, Siyuan Wu, Ricardo Rodríguez-Calvo, Walter Wahli, Manuel Vázquez-Carrera

**Affiliations:** 1 Department of Pharmacology, Toxicology and Therapeutic Chemistry, Faculty of Pharmacy and Food Sciences, University of Barcelona, 08028 Barcelona, Spain; xpalomer@ub.edu (X.P.); jerrywang1258@gmail.com (J.-R.W.); shermantang02@gmail.com (X.T.); wwwwwsy123123@gmail.com (S.W.); 2 Institute of Biomedicine of the University of Barcelona (IBUB), University of Barcelona, 08028 Barcelona, Spain; 3 Spanish Biomedical Research Center in Diabetes and Associated Metabolic Diseases (CIBERDEM)-Instituto de Salud Carlos III, 28029 Madrid, Spain; rrodriguez080480@yahoo.es; 4 Pediatric Research Institute-Hospital Sant Joan de Déu, 08950 Esplugues de Llobregat, Spain; 5 Research Unit on Lipids and Atherosclerosis, University Rovira i Virgili, 43201 Reus, Spain; 6 Vascular Medicine and Metabolism Unit, “Sant Joan de Reus” University Hospital, 43204 Reus, Spain; 7 Institut de Recerca Biomèdica Catalunya Sud, Hospital Universitari Sant Joan de Reus, 43204 Reus, Spain; 8 Center for Integrative Genomics, University of Lausanne, CH-1015 Lausanne, Switzerland; walter.wahli@unil.ch; 9 ToxAlim (Research Center in Food Toxicology), INRAE, UMR1331, F-31300 Toulouse, France

**Keywords:** metabolic dysfunction-associated steatotic liver disease (MASLD), peroxisome proliferator-activated receptor beta/delta (PPARβ/δ), steatosis, inflammation, fibrosis, AMP-activated protein kinase (AMPK), suppressor of mothers against decapentaplegic (SMAD), growth differentiation factor 15 (GDF15)

## Abstract

Metabolic dysfunction-associated steatotic liver disease (MASLD) is considered a hepatic manifestation of insulin resistance and ranges from isolated steatosis to metabolic dysfunction-associated steatohepatitis (MASH). Hepatocyte ballooning, indicative of hepato-cellular damage, and liver inflammation, with or without fibrosis, are characteristic of MASH. Evidence shows that peroxisome proliferator-activated receptor β/δ (PPARβ/δ), expressed in the major liver cells (hepatocytes, Kupffer cells, cholangiocytes, and hepatic stellate cells), may help prevent the progression of MASLD by ameliorating insulin resistance, lipotoxicity, inflammation, and fibrosis. In this review, we summarize the molecular mechanisms by which PPARβ/δ attenuates the progression of MASLD and discuss future research perspectives.

## 1. Introduction

**Figure 1:**
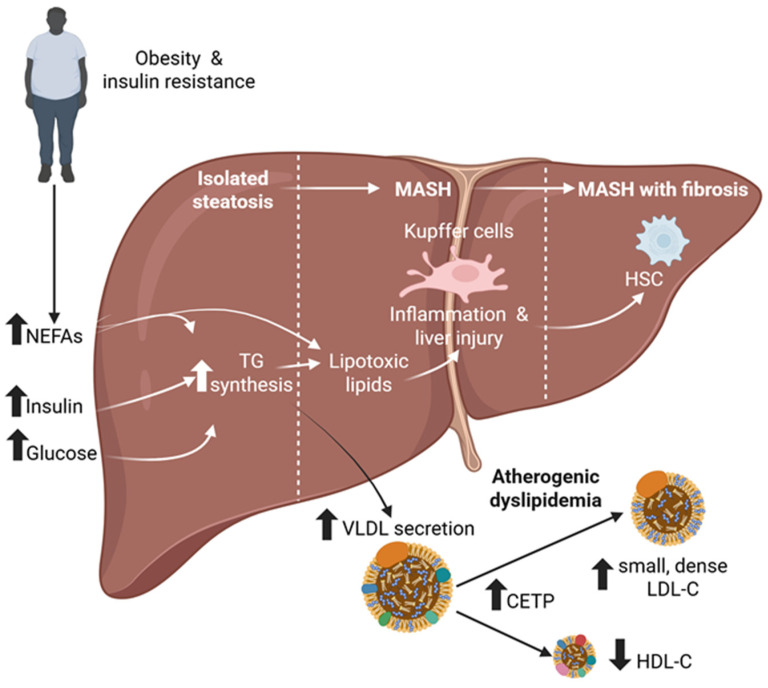
Schematic representation of the pathophysiology of MASLD and atherogenic dyslipidemia. Obesity-associated insulin resistance promotes adipose tissue lipolysis by impairing antilipolytic insulin signaling, leading to increased circulating levels of NEFAs, insulin, and glucose. Excess NEFAs are taken up by the liver, fostering triglyceride accumulation and the development of isolated hepatic steatosis. In steatotic hepatocytes, VLDL secretion is increased, contributing to hypertriglyceridemia and atherogenic dyslipidemia. The intravascular actions of CETP and hepatic lipase promote the formation of small, dense LDL particles and reduce HDL-cholesterol levels. Progressive lipid overload in the liver leads to the generation of toxic lipid species, which induce hepatocellular stress, inflammation, and lipotoxic injury, driving the transition to MASH without fibrosis. Persistent inflammation activates HSCs, resulting in extracellular matrix deposition and progression to MASH with fibrosis. Abbreviations: CETP, cholesteryl ester transfer protein; HDL, high-density lipoprotein; HSCs, hepatic stellate cells; LDL, low-density lipoprotein; MASH, metabolic dysfunction-associated steatohepatitis; MASLD, metabolic dysfunction-associated steatotic liver disease; NEFAs, non-esterified fatty acids; TG, triglyceride; VLDL, very-low-density lipoprotein. Created with BioRender.com.

Metabolic dysfunction-associated steatotic liver disease (MASLD), defined as steatosis plus at least one cardiometabolic risk factor in the absence of harmful alcohol intake, is the leading cause of chronic liver disease and affects 25% of the global population [1,2]. MASLD ranges from steatotic liver disease (fat accumulation in more than 5% of hepatocytes) to progressing to metabolic dysfunction-associated steatohepatitis (MASH) in about one-quarter of patients [3]. MASH is characterized by liver injury, inflammation, and an increased risk of fibrosis, the latter being the strongest predictor of poor prognosis and progression to cirrhosis or hepatocellular carcinoma (HCC) [1]. The main drivers of MASLD are obesity and insulin resistance (Figure 1), with estimates suggesting that approximately 70% to 80% of individuals with obesity and approximately 60% to 70% of those with type 2 diabetes mellitus (T2DM) have MASLD [3,4,5,6]. Among patients with MASLD, cardiovascular disease (CVD) is the leading cause of death, followed by extrahepatic cancers and liver-related complications (e.g., cirrhosis and HCC) [3]. One alteration contributing to the increased cardiovascular risk in patients with MASLD is the presence of atherogenic dyslipidemia (Figure 1). This shift in the lipoprotein pattern is characterized by increased serum triglycerides, reduced high-density lipoprotein (HDL)-cholesterol, and increased small, dense low-density lipoprotein (LDL)-cholesterol particles [7].

The pathogenesis of MASLD is complex and only partly understood, but it involves hepatic lipid accumulation, endoplasmic reticulum (ER)-stress–driven apoptosis, and chronic inflammation [8,9]. The transition from simple steatosis to fibrosis is driven by ongoing, unresolved inflammation triggered by damage-associated molecular signals released during liver cell injury. Inflammatory cytokines, including members of the transforming growth factor β (TGF-β) superfamily, ultimately activate quiescent hepatic stellate cells (HSCs). The activated HSCs then undergo transdifferentiation into proliferative, fibrogenic myofibroblast-like cells, characterized by enhanced synthesis and altered expression of extracellular matrix (ECM) components like collagen and fibronectin [10]. TGF-β1 induces ECM-related genes such as those coding for collagen and α-smooth muscle actin (α-SMA) by activating suppressor of mothers against decapentaplegic (SMAD)-dependent and non–SMAD-dependent pathways [11].

Although all stages of MASLD influence prognosis, fibrosis is the strongest predictor of all-cause and liver-related mortality risk [11]. Despite this, the first drugs approved recently for MASH-related fibrosis, resmetirom and semaglutide, mainly target metabolic pathways rather than fibrogenesis and show only modest, variable improvements in fibrosis [12,13]. Therefore, future drugs for MASLD must decrease not only hepatic steatosis but also the resulting cellular damage, inflammation, and fibrosis. New strategies are needed to address this range of mechanisms by targeting multiple pathways [14]. While pharmacological activation of peroxisome proliferator-activated receptor (PPAR)α and PPARγ has well-established roles in lipid metabolism and insulin sensitivity, respectively, PPARβ/δ uniquely integrates a diverse set of mechanisms to modulate metabolic changes, inflammatory pathways, and fibrosis. This makes PPARβ/δ a relatively underexplored but highly relevant regulator of MASLD development. This review summarizes the molecular mechanisms by which PPARβ/δ slows MASLD progression and discusses future research directions. In addition to reviewing the literature, this article also draws on the authors’ own research and experience in the field.

## 2. PPARβ/δ Structure, Activation, and Expression in the Liver

PPARβ/δ belongs to the nuclear hormone receptor superfamily, which comprises ligand-activated transcription factors. There are three PPAR isotypes: PPARα (NRC1, according to the official nomenclature of PPARs suggested by the Nuclear Receptor Nomenclature Committee), PPARβ/δ (NRC2), and PPARγ (NRC3) [15]. These isotypes regulate genes involved in fatty acid transport and oxidation, lipid and carbohydrate metabolism, vascular function, inflammation, cell proliferation, and cellular senescence [16,17,18,19]. PPARβ/δ is a 441-amino-acid protein with a molecular weight of approximately 49.9 kDa and comprises four functional domains: an N-terminal ligand-independent activation domain, a highly conserved DNA-binding domain, a short hinge region, and a C-terminal ligand-binding domain (LBD). The N-terminal activation function-1 (AF-1) domain confers constitutive transcriptional activity, while activation function-2 (AF-2), located within the C-terminal LBD, is essential for ligand-dependent receptor activation [20,21].

**Figure 2:**
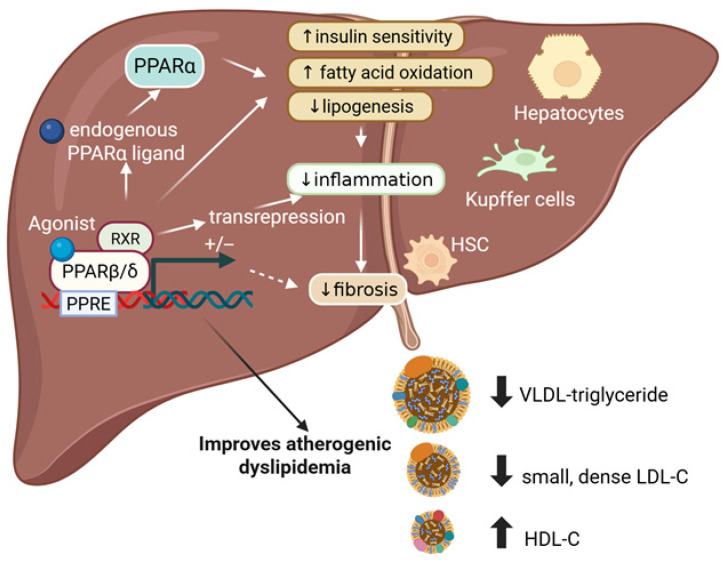
PPARβ/δ improves key pathogenic processes in MASLD. PPARβ/δ functions as a heterodimer with the RXR to regulate gene expression. When activated by a ligand, the PPARβ/δ/RXR complex binds to PPREs in the promoters of target genes, thereby modulating gene transcription. Besides this canonical PPRE-dependent mechanism, PPARβ/δ also exerts anti-inflammatory effects via PPRE-independent transrepression pathways. Pharmacological activation of PPARβ/δ improves insulin sensitivity, decreases lipogenesis and lipotoxicity, reduces inflammation and fibrosis, and corrects atherogenic dyslipidemia. PPARβ/δ activation also increases the availability of endogenous PPARα ligands, supporting coordinated activation of metabolic pathways that further enhance lipid oxidation and promote metabolic homeostasis. Abbreviations: HDL-C, high-density lipoprotein cholesterol; HSCs, hepatic stellate cells; LDL-C, low-density lipoprotein cholesterol; MASLD, metabolic dysfunction-associated steatotic liver disease; PPAR, peroxisome proliferator-activated receptor; PPREs, peroxisome proliferator response elements; RXR, 9-cis retinoic acid receptor; VLDL, very-low-density lipoprotein cholesterol. Adapted from [25]. Created with BioRender.com.

PPARβ/δ functions as a heterodimer with the 9-cis retinoic acid receptor (RXR) and can modulate gene expression through both ligand-dependent and ligand-independent pathways [22]. In the absence of a ligand, PPARβ/δ–RXR complexes associate with corepressor proteins and suppress their transcriptional activity. Ligand binding to the LBD induces a conformational change that releases corepressors and promotes the recruitment of coactivator complexes. The activated PPARβ/δ/RXR heterodimer then binds to peroxisome proliferator response elements (PPREs) in the promoter regions of target genes, driving transcriptional activation [23] (Figure 2). PPARβ/δ can also suppress gene transcription through a PPRE-independent mechanism known as transrepression. Through this mechanism, PPARβ/δ directly interacts with other transcription factors, particularly those that regulate inflammatory genes, preventing their binding to DNA and inhibiting the transcription of their target genes [24]. Transrepression is considered the primary mechanism underlying the anti-inflammatory effects of PPARβ/δ.

Additionally, PPARβ/δ activity can be modulated by posttranslational modifications, including phosphorylation and ubiquitination. Its transcriptional function can be influenced by signaling pathways involving protein kinase A (PKA), phosphatidylinositol 3-kinase (PI3K), and p38 mitogen-activated protein kinase (MAPK) [26,27].

PPARβ/δ is expressed throughout the body, with levels varying among cell types and species. In the mouse liver, PPARβ/δ is highly expressed in HSCs, cholangiocytes, and liver sinusoidal endothelial cells (LSECs), and to a lesser extent in hepatocytes [28,29]. In adult human livers, PPARβ/δ levels are lower than in mouse livers across all hepatic cell types [30,31]. Although PPARβ/δ expression is very low in most human cancer cell lines, human hepatocyte-derived Huh-7 and HepG2 cells respond to PPARβ/δ agonists [32,33]. Moreover, clinical trials have shown that the human liver responds to PPARβ/δ agonists [34,35].

## 3. PPARβ/δ Ligands

PPARβ/δ has a large ligand-binding pocket, enabling it to interact with a wide range of endogenous and synthetic ligands, as well as xenobiotics. Fatty acids and their derivatives are natural low-affinity ligands for the three PPAR isotypes. The development of various PPARβ/δ ligands, such as GW0742, which binds PPARβ/δ with more than a 300-fold greater specificity than either PPARα or PPARγ [36], has significantly advanced our understanding of the functions of this nuclear receptor and facilitated studies on their effects in MASLD. GW501516, with 1000-fold higher specificity for PPARβ/δ over the other two PPAR isotypes, has also shown promising beneficial effects on dyslipidemia and the prevention of T2DM in preclinical models, but further development stopped when mice and rats developed cancer during treatment [37]. It remains unclear whether this carcinogenic effect was due to PPARβ/δ activation itself or due to an off-target effect. Nevertheless, the PPARβ/δ-selective agonist, seladelpar (MBX-8025), and the dual PPARα-β/δ agonist, elafibranor (GFT505), both passed two-year carcinogenicity studies in rats and have been approved for the treatment of primary biliary cholangitis (PBC) in humans. This suggests that tumorigenic activity is not a class effect of PPARβ/δ modulators [38].

## 4. Roles of PPARβ/δ in MASLD

Hepatic PPARβ/δ has diminished mRNA expression and DNA-binding activity in individuals with MASLD [39], and its protein levels are similarly reduced in the livers of *ob/ob* mice and those fed a high-fat diet (HFD) [40,41]. Notably, pharmacological activation of PPARβ/δ ameliorates MASLD across multiple animal models. In the following sections, we discuss how PPARβ/δ influences key pathogenic processes in MASLD (Figure 2).

### 4.1. The Role of PPARβ/δ in Insulin Resistance and Glucose Homeostasis

The hepatic manifestation of insulin resistance is MASLD. Insulin resistance is closely associated with ectopic lipid accumulation [42], leading to adipose tissue failure to respond to insulin’s antilipolytic effect and increased release of non-esterified fatty acids (NEFAs). This state favors liver triglyceride accumulation (Figure 1). Extensive evidence shows an overlap between T2DM and MASLD [43]. T2DM drives the progression of MASLD, accelerating hepatic and extrahepatic adverse outcomes [44], while MASLD increases the likelihood of developing T2DM and has a detrimental effect on glucose metabolism in people with T2DM [45].

The PPARβ/δ agonist GW501516 causes a dose-dependent decrease in fasting insulin level in obese insulin-resistant rhesus monkeys [46] and improves insulin resistance in rats and mice [47,48,49] (Figure 2). GW0742 [50] and elafibranor [51] also attenuate glucose intolerance caused by feeding an HFD in mice. This improved insulin resistance is mediated by increased mitochondrial fatty acid β-oxidation in the liver and other tissues. Consistent with a role for this nuclear receptor in glucose homeostasis, *Ppard* suppression causes glucose intolerance in mice [52].

Additional mechanisms also contribute to the antidiabetic effects of PPARβ/δ. Its activation enhances insulin sensitivity in db/db mice by increasing hepatic glucose metabolism and thereby stimulating the pentose phosphate pathway [52]. Activation of AMP-activated protein kinase (AMPK) by PPARβ/δ agonists also helps improve insulin resistance [53]. AMPK is a key energy sensor activated during low cellular energy states, and enhances fatty acid oxidation and glucose uptake, thereby improving insulin sensitivity. Obesity-induced, chronic, low-grade inflammation may promote insulin resistance. Macrophages have an established role in this inflammatory process, with *Ppard* ablation in macrophages known to disrupt adipose tissue homeostasis and promote insulin resistance [54]. In the liver, interleukin 6 (IL-6) decreases insulin signaling by activating signal transducer and activator of transcription 3 (STAT3), which induces suppressor of cytokine signaling 3 (SOCS3); in turn, this may inhibit the insulin signaling pathway through mechanisms that include insulin receptor substrate (IRS) degradation [33]. PPARβ/δ activation may improve insulin sensitivity in hepatic cells by preventing IL-6-dependent induction of extracellular-related kinase 1/2 (ERK1/2), a serine-threonine protein kinase involved in STAT3 serine phosphorylation [33], or by interacting with T-cell protein tyrosine phosphatase 45 (TCPTP45) [55]. The PPARβ/δ-TCPTP45 interaction blunts IL-6–induced insulin resistance by accelerating TCPTP45-mediated deactivation of the STAT3-SOCS3 signal in a process mediated by sequestration of TCPTP45 within the nucleus. Reduced TCPTP45 translocation into the cytoplasm prevents access to the insulin receptor and, consequently, its inhibitory effect on insulin signaling. Likewise, the PPARβ/δ agonist GW501516 increases insulin receptor β subunit (InsRβ) protein levels in the skeletal muscle of mice [56]. GW501516 also prevents ER stress-mediated reduction of InsRβ levels by reducing ER stress and lysosomal activity. PPARβ/δ activation attenuates InsRβ degradation in lysosomes by reducing the levels of the tyrosine kinase ephrin receptor B4 (EphB4), a protein that binds to InsRβ and facilitates its endocytosis and lysosomal degradation [56].

Finally, a recent study shows that PPARβ/δ is essential for the protective effects of glucagon-like peptide 1 (GLP-1) receptor agonists on mitochondrial function and on the insulin secretion capacity of pancreatic β-cells under lipotoxicity [57]. Overall, these findings suggest that PPARβ/δ improves insulin resistance by coordinating lipid and glucose metabolism in both the liver and extrahepatic tissues.

### 4.2. PPARβ/δ-Mediated Regulation of Lipid Metabolism and Liver Steatosis

Although fibrosis is the strongest predictor of clinical outcomes, steatosis is the initial step that triggers the inflammation that progresses to fibrosis and cirrhosis [58]. Therefore, reducing liver fat is a primary goal in the treatment of MASLD. Lipid storage in the liver, mainly triglycerides from fatty acids, depends on a dynamic balance between fatty acid synthesis, cellular uptake, oxidation, and triglyceride export into very low-density lipoproteins (VLDL). Hepatic fatty acids originate from multiple sources: about 59% from NEFAs, 26% from *de novo* lipogenesis, and 15% from dietary intake [59]. PPARβ/δ activation decreases circulating NEFAs by regulating genes involved in fatty acid oxidation in the liver and other tissues [56,60,61] (Figure 2). Pharmacological activation of PPARβ/δ also promotes fatty acid oxidation via PPARγ coactivator 1α (PGC-1α), a master regulator of mitochondrial biogenesis and function [62]. PPARβ/δ activation may also increase fatty acid oxidation by modulating the cellular location of lipin 1. This protein determines whether fatty acids are incorporated into triglycerides or undergo mitochondrial β-oxidation [62]. In the cytoplasm, lipin 1 is a phosphatidate phosphatase enzyme that promotes triglyceride accumulation and phospholipid synthesis, while in the nucleus, lipin 1 acts as a transcriptional coactivator regulating the induction of PGC-1α-PPARα-target genes involved in fatty acid oxidation. Notably, PPARβ/δ activation in the liver promotes the PGC-1α-lipin 1-PPARα pathway [60]. Therefore, pharmacological activation of PPARβ/δ can stimulate the PPARα-mediated fatty acid oxidation pathway to increase hepatic levels of 16:0/18:1-phosphatidylcholine, an endogenous PPARα ligand that enhances fatty acid utilization in muscle and lowers postprandial lipid levels [60,63] (Figure 2).

Diet-induced liver steatosis can be promoted by phosphatidylcholine transfer protein (PC-TP), which represses PPARβ/δ transcriptional activity in a ligand-dependent manner [64]. Consistent with this observation, hepatic *Pctp* deletion induces PPARβ/δ-regulated transcripts in HFD-fed mice, increasing insulin and glucose sensitivity and decreasing weight gain and lipid accumulation in the liver and skeletal muscle. AMPK activation by PPARβ/δ ligands also stimulates fatty acid oxidation and reduces liver steatosis caused by an HFD [53]. We have also found that the metabolic benefits of pharmacological activation of PPARβ/δ, including reduced liver steatosis, may involve the stress-activated cytokine growth differentiation factor 15 (GDF15). In fact, the positive effects of GW501516 on glucose intolerance, fatty acid oxidation, liver steatosis, ER stress, inflammation, and AMPK activation in HFD-fed mice are abrogated by injecting a GDF15-neutralizing antibody or in Gdf15^−/−^ mice [65]. PPARβ/δ may also increase fatty acid oxidation and mitigate liver steatosis by modulating autophagy, a cellular process key to lipid metabolism. Autophagy is often impaired in MASLD and can lead to increased hepatic lipid accumulation and inflammation [66]. PPARβ/δ stimulates fatty acid β-oxidation and reduces liver steatosis via an autophagy-lysosomal pathway that involves activation of AMPK and reduction of mammalian target of rapamycin (mTOR), two kinases implicated in autophagy regulation [40].

PPARβ/δ activation may attenuate liver steatosis in MASLD by altering de novo lipogenesis from non-lipid sources (Figure 2). Pharmacological PPARβ/δ activation by GW501516 decreases liver fatty acid synthesis by inhibiting *de novo* lipogenesis via an AMPK-mediated pathway [67]. PPARβ/δ activation also inhibits the proteolytic cleavage of sterol regulatory element binding protein-1c (SREBP-1c), a transcription factor regulating genes involved in glycolysis and de novo lipogenesis, via *Insig-1* induction. In a genetic mouse model of MASLD (db/db mice), this process ultimately improves hepatic steatosis [68]. Ppard^−/−^ mice show increased SREBP-1c compared to wild-type animals [69]. Although short treatment with a PPARβ/δ agonist may induce a transient increase in liver triglyceride levels [70], this does not result in hepatotoxicity because PPARβ/δ increases the amount of monounsaturated fatty acids while decreasing the amount of saturated fatty acids [71]. The transient increase in hepatic triglycerides during short-term PPARβ/δ agonist treatment is reversed with longer administration. Over time, the continued induction of fatty acid oxidation, combined with the suppression of de novo lipogenesis, results in a net decrease in hepatic triglyceride accumulation.

The beneficial effects of PPARβ/δ agonists in liver steatosis and MASLD may involve increasing the expression and secretion of hepatokines like fibroblast growth factor 21 (FGF21), which has protective metabolic effects in MASLD. FGF21 improves insulin resistance and reduces hepatic steatosis, lipotoxicity, oxidative stress, ER stress, inflammation, and fibrosis [72]. PPARβ/δ agonists increase circulating FGF21 levels in humans [73], suggesting this hepatokine could mediate the benefits of these compounds.

Collectively, these findings indicate that PPARβ/δ agonists reduce liver steatosis by promoting fatty acid oxidation in the liver and extrahepatic tissues, thereby decreasing the availability of fatty acids to be stored as triglycerides or other complex lipids. This mechanism helps improve liver insulin sensitivity by reducing lipotoxicity. Inhibition of de novo lipogenesis and increased hepatokine secretion also contribute to the effects of PPARβ/δ agonists on liver steatosis.

### 4.3. The Role of PPARβ/δ in Atherogenic Dyslipidemia

In liver steatosis, the secretion of VLDL transporting triglycerides is increased and is the main cause of atherogenic dyslipidemia that characterizes MASLD. This hypertriglyceridemia, combined with the intravascular action of cholesteryl ester transfer protein (CETP) and hepatic lipase, leads to other key features of atherogenic dyslipidemia such as increased small dense LDL-cholesterol particles and reduced HDL-cholesterol levels [74] (Figure 1). Atherogenic dyslipidemia is a significant independent risk factor for CVD, the leading cause of morbidity and mortality in patients with MASH [75].

PPARβ/δ agonists significantly reduce plasma triglyceride levels (Figure 2), and serum triglyceride levels increase in *Ppard*-null mice fed a HFD due to hepatic VLDL overproduction. These effects on serum triglycerides may partly result from the ability of PPARβ/δ to regulate the expression of various genes involved in lipoprotein metabolism, including VldlR, ApoAIV, ApoAV, and ApoCI [76].

PPARβ/δ also raises serum HDL-cholesterol while decreasing LDL-cholesterol levels [46] (Figure 2). The increase seems to result from enhanced production of apolipoproteins ApoAI and ApoAII by the liver. The decrease in LDL-cholesterol is thought to result from decreased levels of the intestinal cholesterol-transport protein Niemann-Pick C1 Like 1 (NPC1L1) [77]. PPARβ/δ activation also promotes the cholesterol elimination in the feces of mice by boosting transintestinal cholesterol efflux [78].

Interestingly, SMAD3 reduces PPARβ/δ levels, which induces atherogenic dyslipidemia and MASLD in db/db mice, with evidence that removing or blocking SMAD3 restores PPARβ/δ levels and improves these conditions [79]. Collectively, these findings confirm a key role for PPARβ/δ in preventing atherogenic dyslipidemia.

### 4.4. PPARβ/δ in the Progression from Isolated Steatosis to MASH

Lipotoxicity is a key driver of MASLD progression and occurs when lipid accumulation in the liver causes cellular stress responses. In isolated steatotic liver disease, lipid accumulation is usually harmless because inert lipids (e.g., triglycerides and cholesterol esters), dominate. By contrast, there is an increase in more reactive lipid species (e.g., free cholesterol, diacylglycerols, and ceramides) during MASH that actively promotes progression. This lipotoxicity activates stress pathways that cause inflammation and hepatocyte damage. The selective PPARβ/δ agonist seladelpar decreases hepatic storage of lipotoxic lipids, improving MASH pathology in atherogenic diet–fed obese diabetic mice [80]. Similarly, the dual PPARα-β/δ agonist elafibranor is protective against steatosis, inflammation, and fibrosis in various animal models of MASLD/MASH and liver fibrosis [81] (Figure 2). Even without PPARα, elafibranor inhibits Western diet-induced liver steatosis and inflammation, suggesting its protective actions are mediated through PPARβ/δ activation. Supporting this, pharmacological activation of PPARβ/δ decreases inflammatory gene expression in the liver [67,82,83] and lessens hepatic ER stress [67], both of which reduce inflammation. Liver inflammation induced by carbon tetrachloride (CCl_4_) in mice worsens with *Ppard* deficiency, but it is attenuated by the PPARβ/δ agonist GW0742 in wild-type mice [84,85]. This anti-inflammatory action of PPARβ/δ appears to involve immune cells, particularly macrophages, by promoting macrophage polarization. PPARβ/δ stimulates Kupffer cells (liver-resident macrophages) to shift toward an anti-inflammatory M2 state (rather than the classical pro-inflammatory M1 state) and helps maintain hepatic function under IL-4 stimulation. Conversely, loss of *Ppard* in Kupffer cells severely impairs this alternative activation, causing liver dysfunction and insulin resistance [86]. PPARβ/δ agonists activate macrophages to shift into a distinctive anti-inflammatory state by inhibiting nuclear factor κ-light-chain-enhancer of activated B cells (NF-κB) and STAT3, as well as specific immune stimulatory components [87]. Interestingly, PPARβ/δ reduces inflammation and cell damage in hepatic ischemia/reperfusion injury by suppressing NF-κB activity in Kupffer cells [88]. Furthermore, activation of PPARβ/δ inhibits IL-6–induced STAT3 activation by suppressing ERK1/2 phosphorylation and restoring phosphorylated AMPK levels [30]. The PPARβ/δ agonist GW501516 decreases fatty acid-induced inflammation and steatosis by suppressing the activation of nucleotide-binding and oligomerization domain (NOD)-like receptor family pyrin domain-containing 3 (NLRP3) inflammasome [89].

Lastly, PPARβ/δ might influence MASLD development through the gut-liver axis. In this pathway, *Ppard*-null mice fed a HFD develop gut dysbiosis with more intestinal lipopolysaccharide (LPS)-rich bacteria, a significant drop in short-chain fatty acids (SCFA)-producing bacteria, and damage to the intestinal mucosal barrier leading to increased endotoxin delivery and worsened liver inflammation [90]. Overall, these findings indicate that the anti-inflammatory effects of PPARβ/δ primarily involve macrophage polarization, inhibition of pro-inflammatory transcription factors, like NF-κB and STAT, inflammasome activity and modulation of the gut-liver axis.

Hepatocyte injury is the initiating event in liver fibrosis. Ongoing liver damage and profibrotic stimuli lead to the activation of HSCs, which undergo transdifferentiation into proliferative, migratory, and fibrogenic myofibroblast-like cells. These cells secrete ECM proteins, such as collagen I, which gradually accumulate to form fibrotic scar tissue [91]. Studies assessing PPARβ/δ agonists have shown inconsistent effects on liver fibrosis. One study reported that GW501516 promotes HSC proliferation, leading to enhanced fibrotic and inflammatory responses. In this study, the effect of GW501516 was mediated by the increased phosphorylation of p38 and c-Jun N-terminal kinases via the PI3K/protein kinase C-α/β–mixed-lineage kinase-3 signaling pathway [92]. In another study, the PPARβ/δ agonist KD-3010, but not GW501516, reduced CCl_4_-induced liver injury and ECM protein deposition. Additionally, primary hepatocytes treated with KD-3010, but not GW501516, were protected from CCl_4_-induced cell death partly by decreasing reactive oxygen species [93]. The pro-fibrotic cytokine TGF-β1 activates SMAD3 in HSCs, driving the transcription of ECM genes and promoting fibrosis [49]. TGF-β1 also activates ERK1/2, which phosphorylates SMAD3 and enhances its activity. We have reported that the PPARβ/δ agonist GW501516 completely prevents glucose intolerance and peripheral insulin resistance, blocks collagen accumulation in the liver, and reduces the expression of inflammatory and fibrogenic genes in mice fed a choline-deficient HFD (CD-HFD). In LX-2 cells (immortalized activated human HSCs) PPARβ/δ activation counters TGF- β1-induced HSC activation and SMAD3 phosphorylation, while decreasing the levels of SMAD3 coactivator p300 via AMPK activation and inhibition of the ERK1/2 pathway [50]. Collectively, these inconsistent findings suggest that PPARβ/δ activation by different ligands may involve both ligand-specific signaling and context-dependent responses in hepatic fibrosis. Different agonists may induce distinct conformational changes in PPARβ/δ, thereby recruitment of coactivators or corepressors. Consequently, some ligands mainly activate genes related to anti-inflammatory pathways, while others may boost inflammatory or pro-fibrotic mediators in specific experimental conditions. Additionally, the biological context may critically influence PPARβ/δ-mediated outcomes. Importantly, the effects of PPARβ/δ in MASLD/MASH are also highly cell-type dependent. In hepatocytes, PPARβ/δ primarily reduces lipotoxic stress and improves metabolic homeostasis, whereas in Kupffer cells, it promotes anti-inflammatory polarization. In HSC, its activation may differentially regulate fibrogenic responses depending on ligand properties and disease stage. During early steatosis or mild inflammation, PPARβ/δ activation may improve insulin resistance and metabolic stress, and indirectly attenuate fibrotic progression. In contrast, during established fibrosis, PPARβ/δ activation might enhance pro-fibrotic signaling in specific cell types. Further research is needed to determine the ligand-specific properties of PPARβ/δ agonists and the stages of liver fibrosis where they offer the greatest therapeutic benefit.

## 5. PPARβ/δ as a Therapeutic Target for Treating MASLD in Humans

Despite MASLD being the leading cause of chronic liver disease, it remains underdiagnosed and undertreated. Few therapeutic options exist, with only the thyroid hormone receptor activator resmetirom and the GLP-1 receptor agonist semaglutide recently approved for non-cirrhotic MASH with intermediate to advanced fibrosis. Moreover, these drugs show only modest and variable improvements in fibrosis and mainly target metabolic pathways rather than fibrogenesis. These limitations create an urgent unmet clinical need for new pharmacological therapeutic options for MASLD.

In patients with MASLD, PPARβ/δ agonists have shown beneficial effects on hepatic steatosis and metabolic syndrome [35,94,95]. Their actions include improving insulin sensitivity, stimulating fatty acid oxidation, and reducing inflammation and liver fibrosis.

Several clinical trials have been conducted with the dual PPARα-β/δ agonist elafibranor. In the phase 2b GOLDEN-505 trial, treating non-cirrhotic MASH patients with 120 mg elafibranor daily for 52 weeks improved insulin sensitivity, glucose homeostasis, and lipid metabolism, while reducing inflammation. These promising results led to the phase III RESOLVE-IT trial. However, interim analyses prompted Genfit to stop the study in March 2022, because elafibranor failed to show histological improvement or resolution of MASH without worsening fibrosis after 72 weeks of treatment. The lack of histological efficacy observed in the RESOLVE-IT study highlights key translational challenges for dual PPARα-β/δ agonism in MASH. One possible explanation is that systemic metabolic improvements might not have been enough to trigger the cellular remodeling needed to resolve steatohepatitis or fibrosis regression. Additionally, significant patient heterogeneity in MASH likely diluted treatment effects, indicating that metabolically driven subgroups or earlier disease stages may respond better. In June 2024, elafibranor received accelerated approval from the US Food and Drug Administration (FDA) as a new treatment for PBC, either alone or in combination with ursodeoxycholic acid (UDCA), especially for patients who respond inadequately to first-line UDCA therapy. Elafibranor works by lowering bile acid synthesis and decreasing liver inflammation, with clinical trials demonstrating meaningful improvements in key liver biomarkers, including alkaline phosphatase and bilirubin, highlighting its therapeutic potential in PBC [96].

Selective PPARβ/δ agonists have also shown beneficial metabolic and hepatic effects. Treatment with the PPARβ/δ-selective agonist seladelpar in overweight men and women with mixed dyslipidemia resulted in significant reductions in liver enzyme levels, inflammation markers, insulin resistance, and atherogenic dyslipidemia. Additionally, it lowered levels of LDL-cholesterol, non-HDL-cholesterol, ApoB100, high-sensitivity C-reactive protein (hs-CRP), and hepatic triglycerides [94]. Seladelpar may work synergistically with atorvastatin to improve several metabolic parameters in individuals with dyslipidemia [94,97]. Although seladelpar improved MASH in animal models [80], a phase 2 clinical trial (NCT03551522) that started in June 2018 found no significant difference in MRI-PDFF (Magnetic Resonance Imaging-Proton Density Fat Fraction) between the treatment and placebo groups after 12 weeks with 181 patients with MASH. Like elafibranor, seladelpar was approved by the US FDA in August 2024 for treating PBC in adults who have an inadequate response to UDCA or as monotherapy for those who are intolerant to UDCA.

Another PPARβ/δ agonist, GW501516, has been shown to promote a shift in cholesterol toward a less atherogenic lipoprotein profile and to correct several metabolic abnormalities linked to metabolic syndrome [35,98]. In healthy men with normal lipid levels, GW501516 improved postprandial triglyceride clearance after fat intake and increased HDL-cholesterol levels [99].

## 6. Conclusions and Future Research Perspectives

PPARβ/δ activation is a promising approach for preventing hepatic steatosis and slowing the progression to MASH. However, therapies that target only PPARβ/δ in humans do not fully resolve MASH, because the pathogenesis of this complex liver disease involves multiple pathways that are unlikely to be addressed by a single agent alone. This could partly explain the limited therapeutic effects observed with investigational drugs and the numerous clinical trial failures in MASH reported to date [100]. Considering that multiple factors may simultaneously drive disease progression in a single patient, combination therapy represents the next rational step in drug development. Ideally, the mechanisms stimulated by combined treatments should complement each other to produce additive or synergistic effects. In this context, combining PPARβ/δ agonists with drugs that have complementary mechanisms in MASH, including resmetirom, glucagon-like peptide 1 (GLP-1) receptor agonists, and sodium-glucose cotransporter 2 (SGLT2) inhibitors, warrants further investigation. This opens the door to repurposing PPARβ/δ agonists along with other drugs for treating MASLD/MASH. Long-term safety is also a critical consideration, as chronic activation of PPARβ/δ may have unintended effects.

Other aspects should be considered when assessing the efficacy of PPARβ/δ agonists in MASLD. For example, gender differences and diurnal variations in liver PPARβ/δ have been reported in animal models [101]. Therefore, evaluating the impact of human sex differences on PPARβ/δ function and circadian rhythm of the receptor in the liver might be important for developing future MASLD therapies that target PPARβ/δ. Similarly, polymorphisms or epigenetic variations affecting lipid metabolism, inflammation, and fibrogenesis could lead to different therapeutic outcomes. In this context, genetic and epigenetic differences among individuals may significantly influence how patients respond to PPARβ/δ-targeted therapies. Finally, the gut-liver axis and microbiome composition play key roles in regulating hepatic lipid handling and inflammatory pathways [25].

In summary, while targeting PPARβ/δ offers considerable promise, effective therapies for MASLD/MASH are likely to require personalized, multi-targeted approaches.

## Data Availability

No new data were created or analyzed in this study.

## Abbreviations

The following abbreviations are used in this manuscript:

α-SMA	α-smooth muscle actin
AF-1	Activation function-1 domain
AF-2	Activation function-2 domain
ALP	Alkaline phosphatase
AMPK	AMP-activated protein kinase
ApoAI	Apolipoprotein AI
ApoAII	Apolipoprotein AII
ApoAIV-V	Apolipoprotein AIV-V
ApoB100	Apolipoprotein B100
ApoCI	Apolipoprotein CI
CCl_4_	Carbon tetrachloride
CD-HFD	Choline-deficient high-fat diet
CETP	Cholesteryl ester transfer protein
CVD	Cardiovascular disease
ECM	Extracellular matrix
EphB4	Ephrin receptor B4
ER	Endoplasmic reticulum
ERK1/2	Extracellular signal-regulated kinase 1/2
FGF21	Fibroblast growth factor 21
GLP-1	Glucagon-like peptide 1
GDF15	Growth differentiation factor 15
HDL	High-density lipoprotein
HFD	High-fat diet
HSC	Hepatic stellate cell
HCC	Hepatocellular carcinoma
hs-CRP	high-sensitivity C-reactive protein
IL	Interleukin
InsRβ	Insulin receptor beta subunit
IRS	Insulin receptor substrate
LBD	Ligand-binding domain
LDL	Low-density lipoprotein
LPS	lipopolysaccharide
LSECs	liver sinusoidal endothelial cells
MAPK	Mitogen-activated protein kinase
MRI	Magnetic resonance imaging
MASH	Metabolic dysfunction–associated steatohepatitis
MASLD	Metabolic dysfunction–associated steatotic liver disease
mTOR	Mammalian target of rapamycin
NEFAs	Non-esterified fatty acids
NLRP3	nucleotide-binding and oligomerization domain (NOD)-like receptor family pyrin domain-containing 3
NF–κB	Nuclear factor kappa-light-chain-enhancer of activated B cells
NPC1L1	Niemann-Pick C1-Like 1 protein
PBC	primary biliary cholangitis
PC-TP	Phosphatidylcholine transfer protein
PGC-1α	PPAR-γ coactivator 1 alpha
PI3K	Phosphatidylinositol 3-kinase
PKA	protein kinase A
PPAR	Peroxisome proliferator-activated receptor
PPRE	Peroxisome proliferator response element
RXR	Retinoid X receptor
SREBP-1c	Sterol regulatory element binding protein 1c
SCFA	Short-chain fatty acids
SMAD	Suppressor of mothers against decapentaplegic
SOCS3	suppressor of cytokine signaling 3
STAT	Signal transducer and activator of transcription
TCPTP45	T-cell protein tyrosine phosphatase 45
T2DM	Type 2 diabetes mellitus
TG	Triglyceride
TGF-β	Transforming growth factor beta
UDCA	Ursodeoxycholic acid
VLDL	Very low-density lipoprotein
